# Neutralising reactivity against SARS-CoV-2 Delta and Omicron variants by vaccination and infection history

**DOI:** 10.1186/s13073-022-01066-2

**Published:** 2022-06-10

**Authors:** Enrico Lavezzo, Monia Pacenti, Laura Manuto, Caterina Boldrin, Margherita Cattai, Marco Grazioli, Federico Bianca, Margherita Sartori, Federico Caldart, Gioele Castelli, Michele Nicoletti, Eleonora Nieddu, Elisa Salvadoretti, Beatrice Labella, Ludovico Fava, Maria Cristina Vanuzzo, Vittoria Lisi, Maria Antonello, Carmela Ileana Grimaldi, Chiara Zulian, Claudia Del Vecchio, Mario Plebani, Andrea Padoan, Daniela Maria Cirillo, Alessandra R. Brazzale, Giovanni Tonon, Stefano Toppo, Ilaria Dorigatti, Andrea Crisanti

**Affiliations:** 1 Department of Molecular Medicine, University of Padova, Padova, Italy; 2 Azienda Ospedale Padova, Padova, Italy; 3 Gastroenterology Unit, Department of Medicine, Verona B. Roma University Hospital, Verona, Italy; 4 Department of Cardiac, Thoracic, Vascular Sciences and Public Health, University of Padova, Padova, Italy; 5 Department of Surgery, Oncology and Gastroenterology, University of Padova, Padova, Italy; 6 Paediatrics Unit, Mother and Child Hospital, Surgery, Dentistry, Maternity and Infant Department, Verona University Hospital, Verona, Italy; 7 Neurology Unit, Department of Clinical and Experimental Sciences, University of Brescia, Brescia, Italy; 8 Department of Medicine, University of Padova, Padova, Italy; 9 Emerging Bacterial Pathogens Unit, Division of Immunology, Transplantation and Infectious Diseases, IRCCS San Raffaele Scientific Institute, Milan, Italy; 10 Department of Statistical Sciences, University of Padova, Padova, Italy; 11 Center for Omics Sciences, IRCCS Ospedale San Raffaele, Milan, Italy; 12 Functional Genomics of Cancer Unit, Division of Experimental Oncology, IRCCS San Raffaele Scientific Institute, Milano, Italy; 13 MRC Centre for Global Infectious Disease Analysis and Jameel Institute, School of Public Health, Imperial College London, London, UK; 14 Department of Life Sciences, Imperial College London, London, UK

**Keywords:** SARS-CoV-2, COVID-19, Antibody persistence, Neutralising antibodies, Delta variant, Omicron variant, Vaccination

## Abstract

**Background:**

The continuous emergence of SARS-CoV-2 variants of concern (VOC) with immune escape properties, such as Delta (B.1.617.2) and Omicron (B.1.1.529), questions the extent of the antibody-mediated protection against the virus. Here we investigated the long-term antibody persistence in previously infected subjects and the extent of the antibody-mediated protection against B.1, B.1.617.2 and BA.1 variants in unvaccinated subjects previously infected, vaccinated naïve and vaccinated previously infected subjects.

**Methods:**

Blood samples collected 15 months post-infection from unvaccinated (*n*=35) and vaccinated (*n*=41) previously infected subjects (Vo’ cohort) were tested for the presence of antibodies against the SARS-CoV-2 spike (S) and nucleocapsid (N) antigens using the Abbott, DiaSorin, and Roche immunoassays. The serum neutralising reactivity was assessed against B.1, B.1.617.2 (Delta), and BA.1 (Omicron) SARS-CoV-2 strains through micro-neutralisation. The antibody titres were compared to those from previous timepoints, performed at 2- and 9-months post-infection on the same individuals. Two groups of naïve subjects were used as controls, one from the same cohort (unvaccinated *n*=29 and vaccinated *n*=20) and a group of vaccinated naïve healthcare workers (*n*=61).

**Results:**

We report on the results of the third serosurvey run in the Vo’ cohort. With respect to the 9-month time point, antibodies against the S antigen significantly decreased (*P*=0.0063) among unvaccinated subjects and increased (*P*<0.0001) in vaccinated individuals, whereas those against the N antigen decreased in the whole cohort. When compared with control groups (naïve Vo’ inhabitants and naïve healthcare workers), vaccinated subjects that were previously infected had higher antibody levels (*P*<0.0001) than vaccinated naïve subjects. Two doses of vaccine elicited stronger anti-S antibody response than natural infection (*P*<0.0001). Finally, the neutralising reactivity of sera against B.1.617.2 and BA.1 was 4-fold and 16-fold lower than the reactivity observed against the original B.1 strain.

**Conclusions:**

These results confirm that vaccination induces strong antibody response in most individuals, and even stronger in previously infected subjects. Neutralising reactivity elicited by natural infection followed by vaccination is increasingly weakened by the recent emergence of VOCs. While immunity is not completely compromised, a change in vaccine development may be required going forward, to generate cross-protective pan-coronavirus immunity in the global population.

**Supplementary Information:**

The online version contains supplementary material available at 10.1186/s13073-022-01066-2.

## Background

Understanding the extent and duration of protection developed upon natural SARS-CoV-2 infections and vaccination is a current research priority. Evidence suggests that more than 90% of COVID-19 patients seroconvert after natural infection and develop variable levels of neutralising antibodies [1–3], and demonstrates that the currently European Medicine Agency (EMA) and Food and Drug Administration (FDA) approved vaccines induce humoral and cellular immunity in most individuals [4–7]. However, antibody titres have been reported to wane over time [8–11]. Although memory B cells and cellular immunity can offer a quick and potent response in case of re-exposure to the virus [12], preventing re-infections [11, 13–17] and offering long-term protection regardless of the presence of antibody-escaping mutations [18–20], the interplay between antibody and cellular immunity, and the variation of naturally- and vaccine-acquired protection, remains to be fully characterised and understood. From an immunological perspective, there can be significant differences in the immune response generated by vaccines in individuals who were not infected with SARS-CoV-2 before vaccination, and in subjects who recovered from a natural infection (so-called hybrid immunity). Recent studies have reported of increased potency of ‘hybrid immunity’ [18, 21, 22], with viral antigen persistence in some tissues being hypothesised as a potential mechanism driving the process of memory B and T cell maturation, resulting in an increased affinity against viral antigens.

Long-term immunity against SARS-CoV-2 infection is jeopardised by the continuous evolution of the virus, which has led to the emergence of new strains with increased transmissibility or capable of partially escaping the immune protection elicited from infection and vaccination, thus posing further challenges for epidemic control. The most widespread variants currently circulating are called Omicron strains and belong to a group of sub-lineages of the B.1.1.529 variant of concern (VOC), which subverted the previously circulating B.1.617.2 (Delta) variant [23, 24]. The evolution of new VOCs characterised by changes in the spike protein causing an increasing escape from the immunity generated by previous infections and vaccination, as reported by several studies for the most recent Delta [25–27] and Omicron [26, 28, 29] VOCs, requires continuous epidemiological surveillance to detect the emergence of new variants and monitor the effectiveness of the deployed vaccines.

To investigate the interaction of natural immunity and vaccination in inducing protective immunity, in June 2021 we conducted a serological and viral neutralisation study on a highly characterized cohort of subjects infected during the first wave, back in February 2020. This study follows on from the previous serosurveys conducted in the same population at two and nine months after the initial SARS-CoV-2 outbreak [8, 30], and provides unique longitudinal data on the magnitude, neutralising ability, and persistence of the antibody response against the spike (S) and nucleocapsid (N) antigens of a B.1 SARS-CoV-2 strain circulating at the start of the pandemic, a B.1.617.2 strain circulating in 2021 and the currently circulating BA.1 strain in unvaccinated infected subjects, as well as individuals vaccinated after infection or when naïve.

## Methods

### Laboratory methods

#### Oro-nasopharyngeal swabs

Swab tests were performed as previously described [8, 30]. Briefly, swabs were inserted into the posterior pharynx first, rubbed over tonsillar pillars and posterior oropharynx and then over the nasal wall in the nostrils. SARS-CoV-2 genome was searched with an in-house real-time RT–PCR method targeting the envelope gene (E), according to Corman et al. [31]

#### Serum antibodies detection

The presence of IgG anti-SARS-CoV-2 was investigated in venous blood collected in 5 ml BD Vacutainer Serum Separation Tubes (SST) and centrifuged for 10 min at 1000–1300 RCF (g). Serological tests were performed by trained laboratory staff using the same commercial kits employed in previous serosurveys [8] and produced by Abbott [32], DiaSorin [33], and Roche [34], applying the detection thresholds provided by the manufacturer (Table 1). For DiaSorin, both the new TrimericS and the previous S1/S2 kits were used for comparison.

**Table 1:** Commercial assays employed in the study to identify IgG anti-SARS-CoV-2 antibody levels

Test	Manufacturer	Recognised antigen	Method	Manufacturers’ thresholds
**LIAISON®** **SARS-CoV-2 S1/S2 IgG**	DiaSorin	S1/S2	CLIA^a^	Negative: <12.0 AU/mL Equivocal: 12.0 ≤ × <15.0 AU/mL Positive: ≥15.0 AU/mL
**Elecsys®** **Anti-SARS-CoV-2**	Roche	N	ECLIA^b^	Positive: <1.0 Negative: ≥1.0
**ARCHITECT®** **SARS-CoV-2 IgG**	Abbott	N	CMIA^c^	Negative: <1.4 Positive: ≥1.4
**LIAISON®** **SARS-CoV-2 TrimericS IgG**	DiaSorin	Trimeric S	CLIA^a^	Negative: <33.8 BAU/mL Positive: ≥33.8 BAU/mL

*CLIA* chemiluminescent immunoassay, *ECLIA* electrochemiluminescent immunoassay, *CMIA* chemiluminescent microparticle immunoassay, *AU* arbitrary concentration units, *BAU* binding antibody unit

#### Micro-neutralisation assay

Three independent assays were set up in parallel to assess the neutralisation ability of patients’ serum antibodies against three viral isolates, a third passage B.1 strain isolated in March 2020 (GenBank accession MW468415), a third passage B.1.617.2 strain from August 2021 (GenBank accession OM202516), and a third passage BA.1 strain isolate from February 2022 (GenBank accession ON062195). Heat-inactivated serum samples (30 min at 56 °C) were diluted 1:10 with Dulbecco’s modified Eagle’s medium (DMEM, Gibco™, ThermoFisher) FBS Free medium. 50 μl of viral isolate, diluted in DMEM FBS Free to the final concentration of 100 median tissue culture infective dose (TCID50), were mixed with an equal volume of two-fold serial dilutions of sera in 96-wells microplates and incubated for 1 h at 37 °C in a humidified atmosphere with 5% CO_2_. Following incubation, 100 μL of VERO E6 cells (ATCC® CRL 1586™) suspended in DMEM 6% FBS were added to each well and incubated at 37°C. To titrate neutralising antibodies against the BA.1 isolate, the immunocomplexes were used to infect adherent VERO E6 at 70-80% confluence in a 96-well plate. After 72 h, cytopathic effect was assessed; the supernatant was removed and 120 μl of 5% formaldehyde Gram’s crystal violet 40% m/v were added to each well, followed by 30 min of incubation. After a washing step with water, plates were allowed to dry and the absorbance was read at 595 nm. The neutralisation titre was determined as the highest serum dilution showing an optical density (OD) of 90% or more with respect to the control sera.

### Definition of COVID-19 recovered patients (ground truth, GT)

**Fig. 1:**
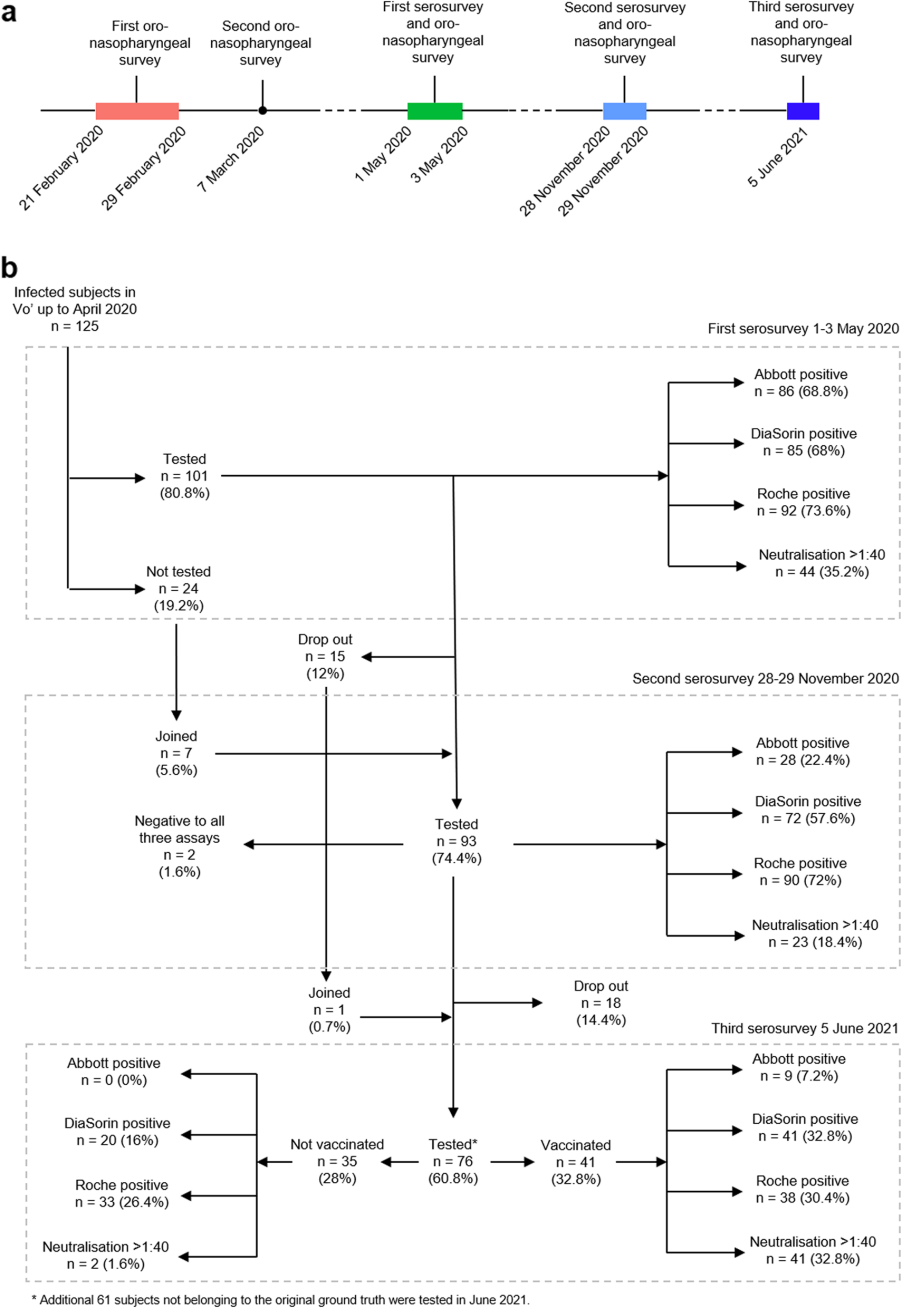
Study description. **a** Timeline of the surveys conducted in the study area since the start of the SARS-CoV-2 epidemic in Vo’. **b** Flow chart illustrating the study design, which focuses on the subjects who were found to be positive early in the pandemic (from February 2020 to May 2020, according to the ground truth definition). The serosurveys were conducted in Vo’ on three time points, 1st–3rd May 2020, 28th–29th November 2020, and 5th June 2021

Multiple rounds of mass testing, that included oropharyngeal swabs and serological assays, allowed for the identification of all the residents in the municipality of Vo’ who were infected and recovered from SARS-CoV-2 infection during the first wave, between February 2020 and March 2020 [8]. To be included among COVID-19 recovered individuals, one of the following criteria had to be satisfied: (i) a positive swab in February 2020 or March 2020, (ii) a viral neutralisation titre greater than 1:40 in May 2020, or (iii) serum reactivity against two serological tests with different antigen targets in May 2020. We refer to this group as baseline ground truth (GT). It included 125 subjects, a size that perfectly fitted the seroprevalence estimated through a multinomial likelihood model [8]. These subjects were followed up at several time points to monitor the presence and persistence of antibodies against both the spike (S) and the nucleocapsid (N) antigens (Fig. 1), as well as to investigate the presence of virus neutralising antibodies (Tables 1 and 2). We previously reported that all subjects belonging to the ground truth were positive to at least one serological assay in May 2020, about two months after the time of their infection [8] (Fig. 1). On occasion of a second serological survey conducted in November 2020, 98.8% of the subjects identified as infected using the ground truth definition were still positive nearly 9 months after the infection [8], although with strong differences depending on the test.

**Table 2:** Observed positivity rates by assays across the three serosurveys, stratified by vaccination status and dose for June 2021

Test	Detected antigen	Positive May 2020 (%)	Positive November 2020 (%)	Positive June 2021 (%)		
				Unvaccinated (%)	1 dose (%)	2 doses (%)
**Abbott**	N	86/92 (93,5)	28/93 (30,1)	0/76 (0)	3/76 (3,9)	6/76 (7,9)
**DiaSorin**	S	85/101 (84,2)	72/93 (77,4)	20/35 (57,1)	21/21 (100)	20/20 (100)
**Roche**	N	92/92 (100)	90/93 (96,8)	33/76 (43,4)	19/76 (25,0)	19/76 (25,0)
**Neutralisation**	S	44/98 (44,9)	23/93 (24,7)	2/35 (5,7)	21/21 (100)	20/20 (100%)

### Cohorts

76 subjects belonging to the GT were tested again in June 2021 to investigate the persistence of antibodies against the SARS-CoV-2 S and N antigens using the Abbott, DiaSorin S1/S2, DiaSorin TrimericS, and Roche immunoassays. The serum neutralising reactivity was also assessed against B.1, B.1.617.2 (Delta), and BA.1 (Omicron) SARS-CoV-2 strains through a micro-neutralisation test. The availability of the results obtained with two different DiaSorin tests on the same individuals allowed to calculate the correlation between the two assays and the conversion factor. 65 of these subjects were tested for anti-N antibodies also in May 2020 and November 2020 and they were used to investigate the N-antigen antibody dynamics 15-months after the infection. The antibody decay rate was calculated only for subjects positive to N-antigen antibodies in May 2020 and with no doubling antibody titres between May and November 2020 or between November 2020 and June 2021 (Abbott *n*=61, Roche *n*=53).

41 out of 76 subjects were vaccinated at the time of sampling: 21 had received only one dose of vaccine (Oxford-AstraZeneca=10; Pfizer-BioNTech=11) at least seven days before the serosurvey, while 20 had received two doses of vaccine (Pfizer-BioNTech=19; Moderna=1) at least seven days before the serosurvey. To monitor S-antigen antibody dynamics at 15-months post infection, only unvaccinated subjects who were tested for anti-S antibodies also in May 2020 and November 2020 (*n*=33) were considered. Anti-S antibody decay rate was calculated for subjects positive to anti-S antibodies in May 2020 and with no doubling antibody titres between May and November 2020 or between November 2020 and June 2021 (DiaSorin *n*=27, micro-neutralisation *n*=9). Vaccinated subjects present in all serosurveys (n=38) allowed us to investigate the effect of vaccination on previously infected subjects. Neutralising antibodies dynamic was assessed on all the subjects who underwent the microneutralisation test also in May 2020 and November 2020 (70 out of 76).

A cohort of 50 naïve individuals was enrolled from the subjects not belonging to the GT (n=30 unvaccinated and n=20 vaccinated). While the unvaccinated naïve subjects were used as a control group, the naïve subjects vaccinated with one dose (*n*=6; Pfizer-BioNTech=2; Oxford-AstraZeneca=4) or 2 doses (*n*=14; Pfizer-BioNTech=13; Oxford-AstraZeneca=1) were used to compare hybrid immunity to vaccine-induced immunity.

Finally, an independent cohort of naïve vaccinated healthcare workers (*n*=61, all with two doses of Pfizer-BioNTech) was enrolled. They were tested with the Diasorin TrimericS and the micro-neutralisation assay to compare hybrid immunity versus vaccine-induced immunity.

Age and vaccination status of the different cohorts are reported in the Additional file 1: Fig.S4.

### Statistical analyses

The antibody decay rate was estimated at the individual level as the logarithmic change in antibody values observed between May 2020 and June 2021 (within the same subject) divided by the number of days between the two serosurveys (400 days). The antibody half-life was estimated as the natural logarithm of 0.5 divided by the antibody decay rate and was calculated on all subjects testing positive in May 2020 serosurvey and without doubling antibody levels in November 2020 and June 2021 (Abbott *n*=65, DiaSorin *n*=29, Roche *n*=53, neutralisation *n*=9). Differences in antibody and neutralisation titres of sera tested against B.1, B.1.617.2 and BA.1 viral strains were assessed with Friedman test for three or more paired samples followed by Dunn’s multiple comparisons test. The same tests were performed when comparing the antibody and neutralisation titres of the same subjects at 2, 9 and 15 months from the infection. For comparisons among groups of unpaired subjects (e.g. according to vaccination or infection status) we used Kruskal-Wallis test followed by Dunn’s multiple comparisons test. The associations between antibody levels and symptom occurrence, hospitalisation, sex, age-group and BMI and between vaccination and pre-exposure, symptom occurrence and sex were assessed using the Kruskal-Wallis test. We used Fisher’s exact test to assess the association between vaccination and previous hospitalisation.

## Results

### Serum reactivity to spike (S) and nucleocapsid (N) antigens

In June 2021, 76 subjects infected by SARS-CoV-2 in February/March 2020 (as defined by the ground truth definition, see the “Methods” section) were tested with the same methods applied in the previous surveys (Methods) (Fig. 1). Overall, all 76 (100%, 95% confidence interval (CI) 95.3–100%) individuals tested positive to at least one assay, with 9 (11.8%, 95% CI 5.6–21.3%) being positive to all three of them. As seen in our previous surveys [8], in June 2021 we observed strong differences in the proportion of positive subjects depending on the assay used, with 11.8% (9 out of 76, 95% CI 5.6–21.3%), 80.3% (61 out of 76, 95% CI 69.5–88.5%), and 93.4% (71 out of 76, 95% CI 85.3–97.8%) testing positive at the 15 months follow up for Abbott, DiaSorin and Roche, respectively. In June 2021, neutralising titres greater than 1:40 were found in 56.6% (43 out of 76, 95% CI 44.7–67.9%) of subjects. Of additional 61 volunteering subjects, who took part in the June 2021 survey and were not identified as infected in February/March 2020 according to our ground truth definition, 3 had a positive swab between May 2020 and December 2020, and 8 showed positivity to at least two different serological assays and were excluded from the analyses; the remaining 50 subjects were used as a naïve control group.

### Impact of vaccination on antibody reactivity

**Fig. 2:**
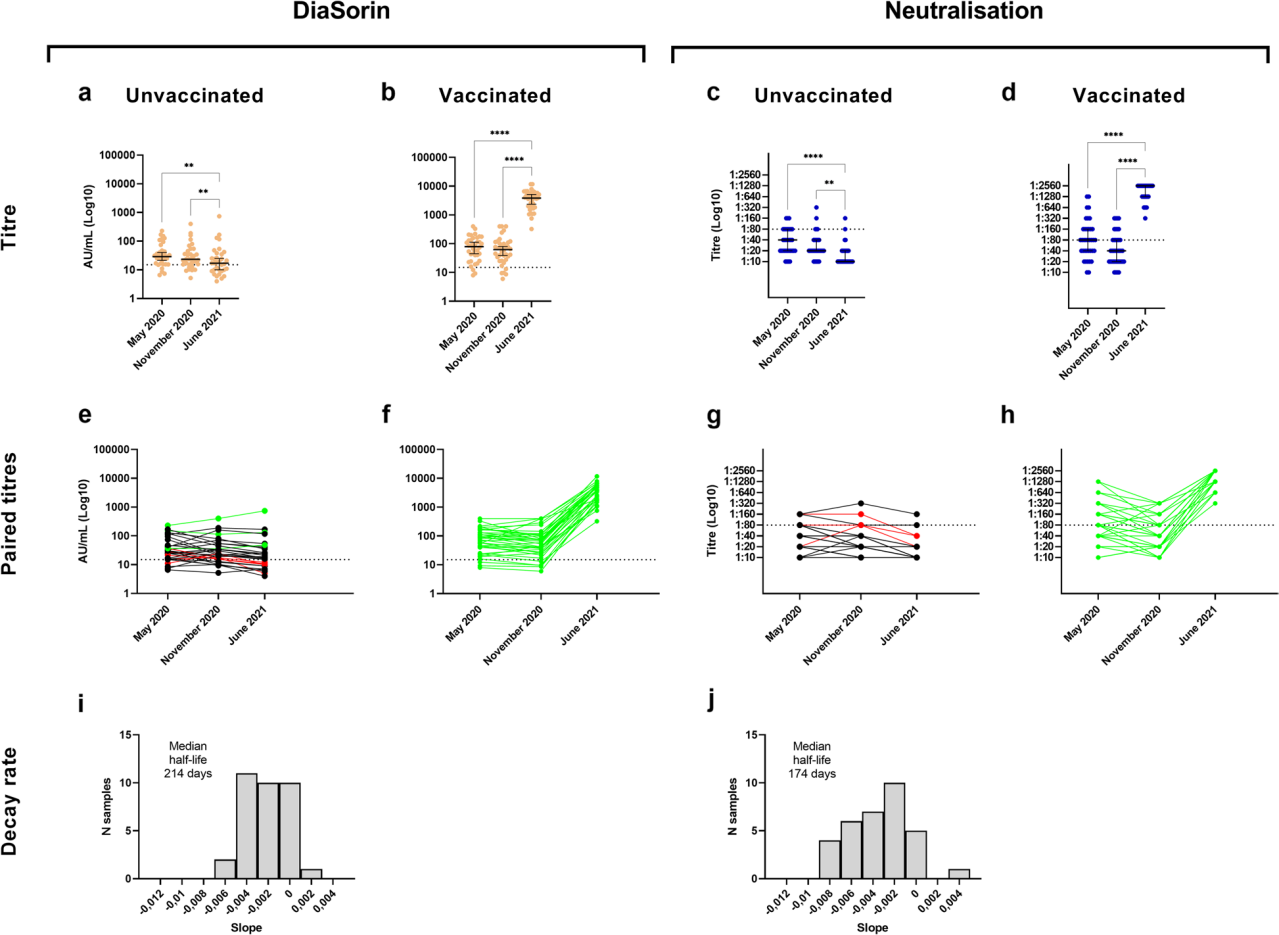
Anti-S antibody titres and dynamics in vaccinated and unvaccinated subjects previously infected by SARS-CoV-2. **a**–**d** Observed antibody titres in unvaccinated and vaccinated subjects infected by SARS-CoV-2 and tested in May 2020, November 2020 and June 2021 by DiaSorin (vaccinated *n*=38, *P* < 0.0001 from November 2020 to June 2021; unvaccinated *n*=33, *P* = 0.0063 from November 2020 to June 2021) and micro-neutralisation assays (vaccinated *n*=38, *P* < 0.0001 from November 2020 to June 2021; unvaccinated *n*=32, *P* = 0.0053 from November 2020 to June 2021). The horizontal line represents the median, the vertical line represents the 95% confidence intervals. **e**–**h** Observed individual-level paired antibody titres in subjects infected by SARS-CoV-2 and tested in May 2020, November 2020 and June 2021. In June 2021, 59.4% (19 out of 32 unvaccinated subjects, 95% CI 40.6–76.3%) and 6.3% (2 out of 32 unvaccinated individuals, 95% CI 0.8–20.8%) had antibodies more than 15 months post infection according to DiaSorin and micro-neutralisation, respectively. Subjects with increasing titres are coloured in green, while subjects with a negative result in June 2021 are presented in red. **i**, **j** Estimated antibody decay rate distributions calculated among the unvaccinated subjects infected by SARS-CoV-2 in February/March 2020, tested in May 2020, November 2020 and June 2021. Each bar represents the frequency of each slope (in units of days), calculated on the logarithm of individual-level sequential titres. We estimated a median half-life of 214 (95% CI 168–288) days and 174 (95% CI 146–202) days for the antibodies detected by the DiaSorin and micro-neutralisation assays, respectively. To estimate the median half-life, only subjects with no doubling antibodies from May 2020 were considered. Asterisks indicate *P < 0.05, **P < 0.01, ***P < 0.001, ****P < 0.0001. Statistical significance of antibody levels was evaluated by Friedman test followed by Dunn’s multiple comparisons test

**Fig. 3:**
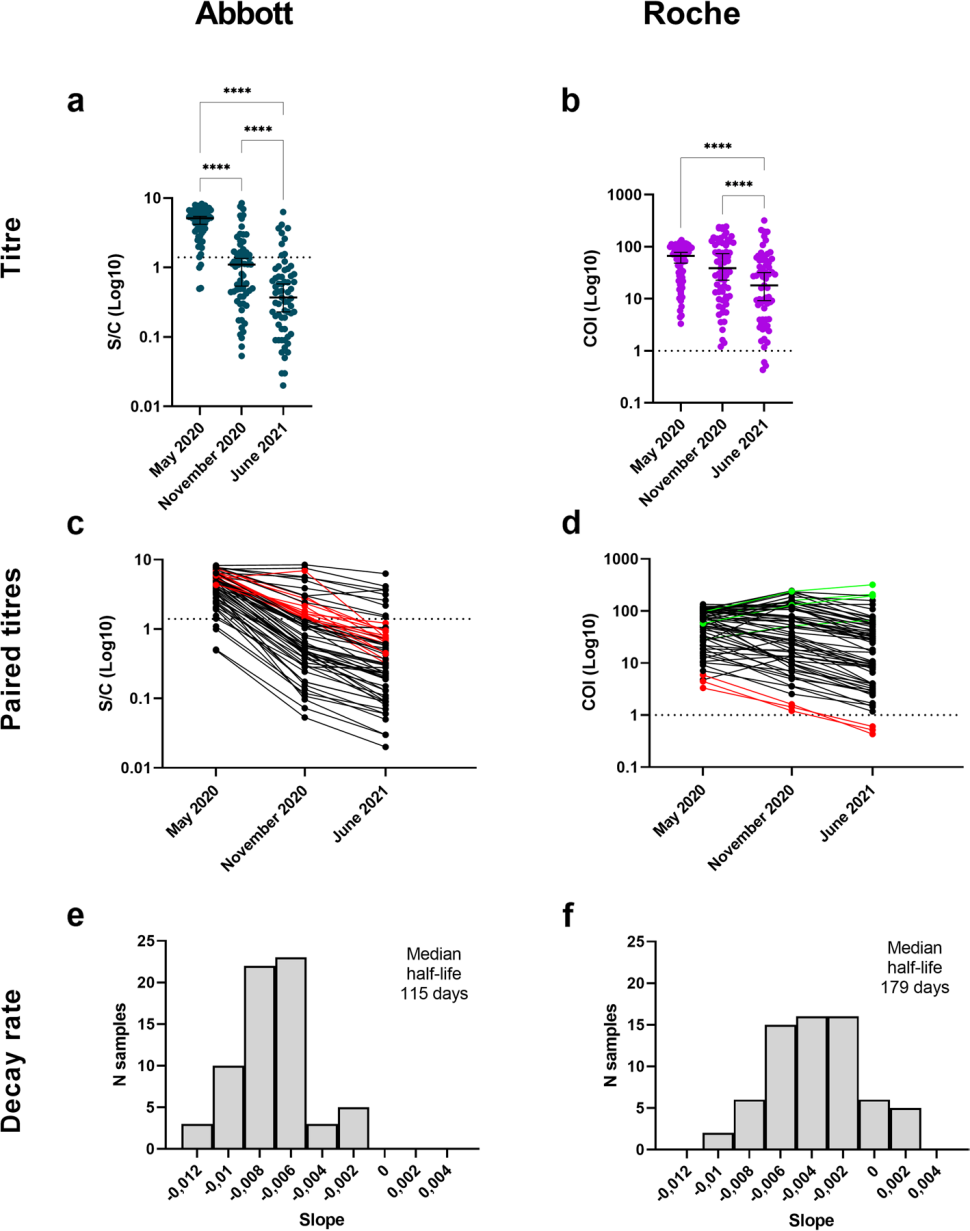
Anti-N antibody titres and dynamics in subjects previously infected by SARS-CoV-2. a-b) Observed antibody titres in subjects infected by SARS-CoV-2 and tested in May 2020, November 2020 and June 2021 with Abbott (*n*=65, *P* < 0.0001 from November 2020 to June 2021) and Roche assays (*n*=65, *P* < 0.0001 from November 2020 to June 2021). The horizontal line represents the median, the vertical line represents the 95% confidence intervals. **c**, **d** Observed individual-level paired antibody titres in subjects infected by SARS-CoV-2 and tested in May 2020, November 2020 and June 2021. In June 2021, 13.8% (9 out of 65 subjects, 95% CI 6.5–24.7%) and 95.4% (62 out of 65 subjects, 95% CI 87.1–99.0) resulted positive to Abbott and Roche assays respectively, more than 15 months post infection. Subjects with increasing titres are coloured in green, and subjects with a negative result in June 2021 are presented in red. **e**, **f** Estimated antibody decay rate distribution calculated among subjects infected by SARS-CoV-2 in February/March 2020 and tested in May 2020, November 2020, and June 2021. Each bar represents the frequency of each slope (in units of days), calculated on the logarithm of individual-level sequential titres. We estimated a median half-life of 115 (95% CI 105–126) days and 179 (95% CI 153–255) days for the antibodies detected by the Abbott and Roche assays, respectively. To estimate the median half-life, only subjects with no doubling antibodies from May 2020 were considered. Asterisks indicate *P < 0.05, **P < 0.01, ***P < 0.001, ****P < 0.0001. Statistical significance of antibody levels was evaluated by Friedman test followed by Dunn’s multiple comparisons test

On 5th June 2021, 53.9% (41 out of 76) of the participants previously infected by SARS-CoV-2 according to the ground truth definition [8] had received at least one dose of spike-based vaccine (either mRNA vaccine or based on adenoviral vector) at least seven days before testing. As expected, vaccination had a strong impact on S-targeting antibody levels (Fig. 2), but not on those directed against the N antigen (Fig. 3). All vaccinated subjects showed reactivity against the S antigen, had a neutralising titre greater than 1:40 (41 out of 41 for both DiaSorin and neutralisation, 95% CI 91.4–100%) (Fig. 2b, d), and they still showed reactivity against the N antigen either when using Abbott (22.0%, 9 out of 41, 95% CI 10.6-37.7%) or Roche (92.7%, 38 out of 41, 95% CI 80.1–98.4%) assays (Additional file 1: Fig. S1b and S1d). In the unvaccinated group, the serum reactivity against the S antigen was significantly lower compared to the vaccinated subjects, with positivity rates of 57.1% (20 out of 35, 95% CI 39.3–73.7%) and 5.7% (2 out of 35, 95% CI 0.7–19.2%) for DiaSorin and micro-neutralisation assays, respectively (Fig. 2a–d). Among 50 naïve subjects, 37.0% (20 out of 54, 95% CI 24.3–51.3%) had received at least one dose of vaccine at least seven days before testing. Of vaccinated naïve subjects, 0% (0 out of 20, 95% CI 0.0–16.9%), 95.0% (19 out of 20, 95% CI 75.1–99.9%), 0% (0 out of 20, 95% CI 0.0–16.9%) and 25% (5 out of 20, 95% CI 8.7–49.1%) were positive to Abbott, DiaSorin, Roche and neutralisation, respectively, whereas 8.8% (3 out of 34, 95% CI 1.9–23.7%), 35.3% (12 out of 34, 95% CI 19.8–53.5%), 2.9% (1 out of 34, 95% CI 0.1–15.3%) and 0% (0 out of 34, 95% CI 0.0–10.3%) of the unvaccinated naïve (as of February/March 2020) subjects showed positivity to the same tests. The unvaccinated naïve subjects with a positive serological test are most likely false positives, since the percentages are in line with our previous positive predictive values estimates for the different assays [8] and the positivity to one test is never confirmed by any of the others.

### Antibody dynamics in vaccinated and unvaccinated subjects

Among vaccinated subjects, independently of the number of doses received, we found a significant increase of both DiaSorin and neutralisation titres (Friedman test followed by Dunn’s multiple comparisons *P* < 0.0001 for both cases), with all subjects showing an increasing trend; on the contrary, in the unvaccinated group, antibodies directed against the S antigen decreased significantly, as measured by DiaSorin and neutralisation (Friedman test followed by Dunn’s multiple comparisons test from November 2020 to June 2021 *P* < 0.01 for both cases) (Fig. 2).

The serum reactivity against the N antigen progressively decreased with time irrespectively of the utilised assay in the whole cohort (Fig. 3, Friedman test followed by Dunn’s multiple comparisons test *P* < 0.0001 for both Abbott and Roche assays) and among vaccinated and unvaccinated individuals separately (Friedman test followed by Dunn’s multiple comparisons test *P* < 0.001) (Additional file 1: Fig. S1). Nonetheless, we observed a significant difference between anti-N antibody titres detected in June 2021 between vaccinated and unvaccinated individuals (Mann-Whitney test *P* < 0.001 for both Roche and Abbott tests) (Additional file 1: Fig. S2). Considering the subjects tested across all serosurveys conducted in May 2020, November 2020, and June 2021, the median half-life of the antibodies detected by Abbott, DiaSorin, Roche, and neutralisation are of 115 days (95% CI 105–126), 229 days (95% CI 179–288), 179 days (95% CI 146–255), and 174 (95% CI 146–202), respectively.

### Correlation between two DiaSorin assays and neutralisation

We assessed in parallel the performance of two DiaSorin tests, the first version targeting antibodies against the S1/S2 antigen and the updated version containing a full trimeric spike antigen. The two assays showed a strong correlation (Spearman’s *r* = 0.963, 95% CI 0.941-0.977, *P* value < 0.0001) (Additional file 1: Fig. S3) and concordance (Additional file 1: Table S1). We estimated a conversion factor between the two assays of 4.451 (95% CI 4.024–4.878) by linear regression and found a high correlation between the antibody levels measured by the DiaSorin assays and the neutralising titres (DiaSorin S1/S2 vs neutralisation: Spearman’s *r* = 0.954, 95% CI 0.916–0.975; DiaSorin TrimericS vs neutralisation: Spearman’s *r* = 0.940, 95% CI 0.890–0.967; all *P* values are significant, *P* < 0.0001) (Additional file 1: Fig. S3).

### Hybrid immunity provides higher anti-S antibody and neutralisation titres than vaccination in naïve subjects

**Fig. 4:**
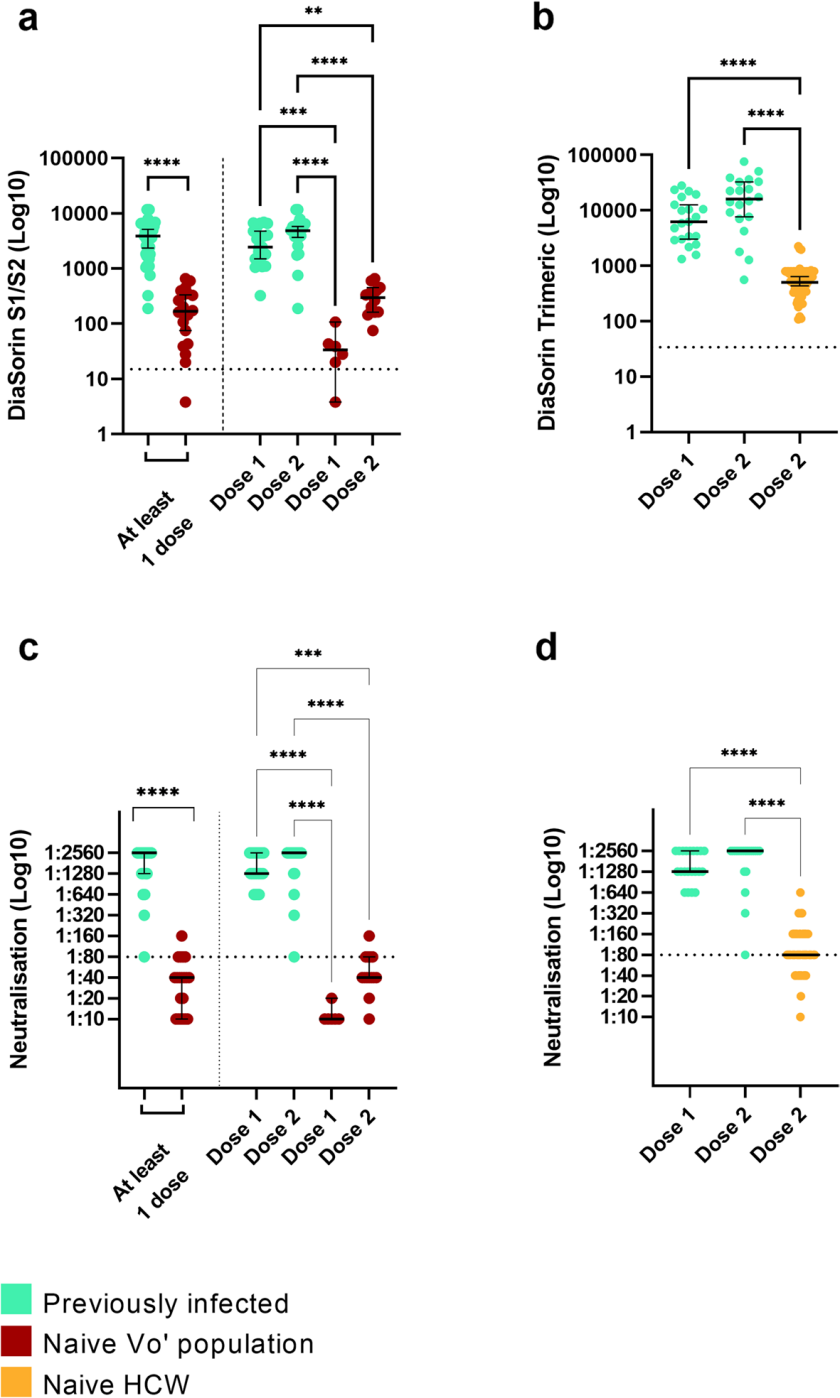
Antibody levels in vaccinated naïve and vaccinated previously infected individuals according to DiaSorin and micro-neutralisation assays. **a**, **b** Observed antibody levels measured by DiaSorin assays in vaccinated naïve and previously infected individuals with at least one dose of vaccine (Mann-Whitney test, *P* < 0.0001) and with one or two doses of vaccine (Kruskal-Wallis test followed by Dunn’s multiple comparisons test, vaccinated naïve versus previously infected subjects after one vaccine dose, *P* < 0.0001; after two vaccine doses, *P* = 0.01; vaccinated naïve HCW versus previously infected subjects after two vaccine doses, *P* < 0.0001). **c**, **d** Observed neutralising antibody titres measured by a micro-neutralisation assay in vaccinated naïve and previously infected individuals with at least one dose of vaccine (Mann-Whitney test, *P* < 0.0001) and with one or two doses of vaccine (Kruskal-Wallis test followed by Dunn’s multiple comparisons test, vaccinated naïve versus previously infected subjects after one or two vaccine doses, *P* < 0.0001; vaccinated naïve HCW versus previously infected subjects after two vaccine doses, *P* < 0.0001). Asterisks indicate *P < 0.05, **P < 0.01, ***P < 0.001, ****P < 0.0001. HCW: healthcare workers. Previous infection is defined according to the ground truth definition provided in the text

We investigated the impact of past SARS-CoV-2 infection to the humoral immune response induced by vaccination (hybrid immunity) as measured by anti-S antibodies and neutralisation titres. Comparing the antibody titres of subjects from the Vo’ cohort (*n* = 20) vaccinated when naïve to vaccinated individuals that were previously infected (*n* = 41) we observed significantly higher titres in vaccinated individuals that were previously infected (Mann-Whitney test, *P* < 0.0001). Even after two vaccine doses, the difference in antibody titres between subjects vaccinated when naïve and subjects vaccinated post infection remains significant (Fig. 4a, Kruskal-Wallis test followed by Dunn’s multiple comparisons test, *P* < 0.0001). Neutralisation and anti-S titres observed in vaccinated subjects that were previously infected after one and two vaccine doses were statistically comparable (Kruskal-Wallis test followed by Dunn’s multiple comparisons test, *P* = 1 for both DiaSorin and neutralisation). Similar trends were observed when comparing the group of vaccinated subjects previously infected with SARS-CoV-2 with an independent cohort of healthcare workers (HCW, *n* = 61) vaccinated when naïve from the complex operational unit (U.O.C.) of Microbiology and Virology of Padua University Hospital (Kruskal-Wallis test followed by Dunn’s multiple comparisons test, *P* < 0.0001 for both DiaSorin and neutralisation) (Fig. 4b, d).

### Two vaccine doses in naïve individuals trigger higher anti-S antibodies and neutralising titres than natural infection

**Fig. 5:**
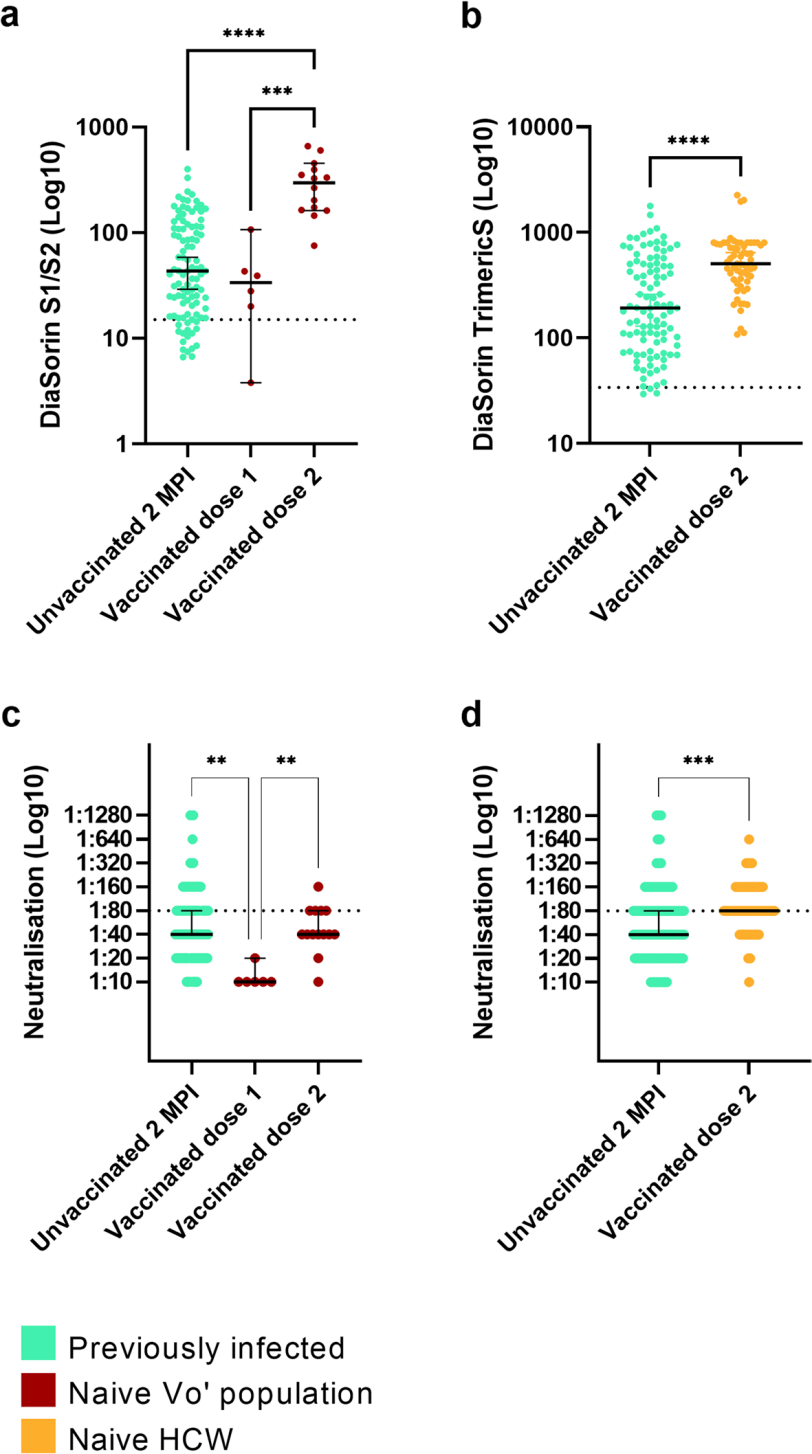
Anti-S antibody levels and neutralisation titres induced by vaccination and natural infection. Observed antibody levels in Vo’ unvaccinated individuals previously infected with SARS-CoV-2, Vo’ subjects and HCW subjects vaccinated when naïve, according to **a** DiaSorin S1/S2 (Kruskal-Wallis test followed by Dunn’s multiple comparisons test, unvaccinated previously infected versus vaccinated when naïve after one dose of vaccine, *P* = 1, or two doses of vaccine, *P* < 0.0001), **b** DiaSorin TrimericS (Mann-Whitney test, unvaccinated previously infected versus HCW subjects vaccinated when naïve, *P* < 0.0001) (**c**, **d**) and micro-neutralisation (Kruskal-Wallis test followed by Dunn’s multiple comparisons test, unvaccinated previously infected versus vaccinated when naïve after one dose of vaccine, *P* = 0.001, or two doses of vaccine, *P* = 1; Mann-Whitney test, unvaccinated previously infected versus HCW subjects vaccinated when naïve, *P* < 0.001) assays. Asterisks indicate *P < 0.05, **P < 0.01, ***P < 0.001, ****P < 0.0001. HCW: healthcare workers. Previous infection is defined according to the ground truth definition provided in the text

Using the conversion factor calculated to convert the results of the old DiaSorin S1/S2 assay into the new DiaSorin trimericS assay, we compared the antibody response after natural infection with the response to vaccination, roughly two months after the immune stimulus. Vo’ subjects infected by SARS-CoV-2 in February/March 2020 and tested in May 2020 showed lower anti-S antibody levels with respect to both naïve subjects from Vo’ (Kruskal-Wallis test followed by Dunn’s multiple comparisons test, *P* < 0.0001) and HCW (Mann-Whitney test, *P* < 0.0001) after two doses of vaccine (Fig. 5a, b). A similar trend was observed for neutralising antibody titres, although the difference is significant only between previously infected subjects and vaccinated HCW (Mann-Whitney test, *P* < 0.001) (Fig. 5c, d).

### Association analysis

Among infected unvaccinated subjects, we observed no significant differences in the antibody titres by symptom occurrence, hospitalisation, sex, age-group and BMI. In the Vo’ cohort, we observed no statistically significant difference in the number of vaccinated and unvaccinated subjects by infection status (according to the baseline ground truth definition), symptom occurrence and sex.

### Neutralisation reactivity of Delta and Omicron VOCs

**Fig. 6:**
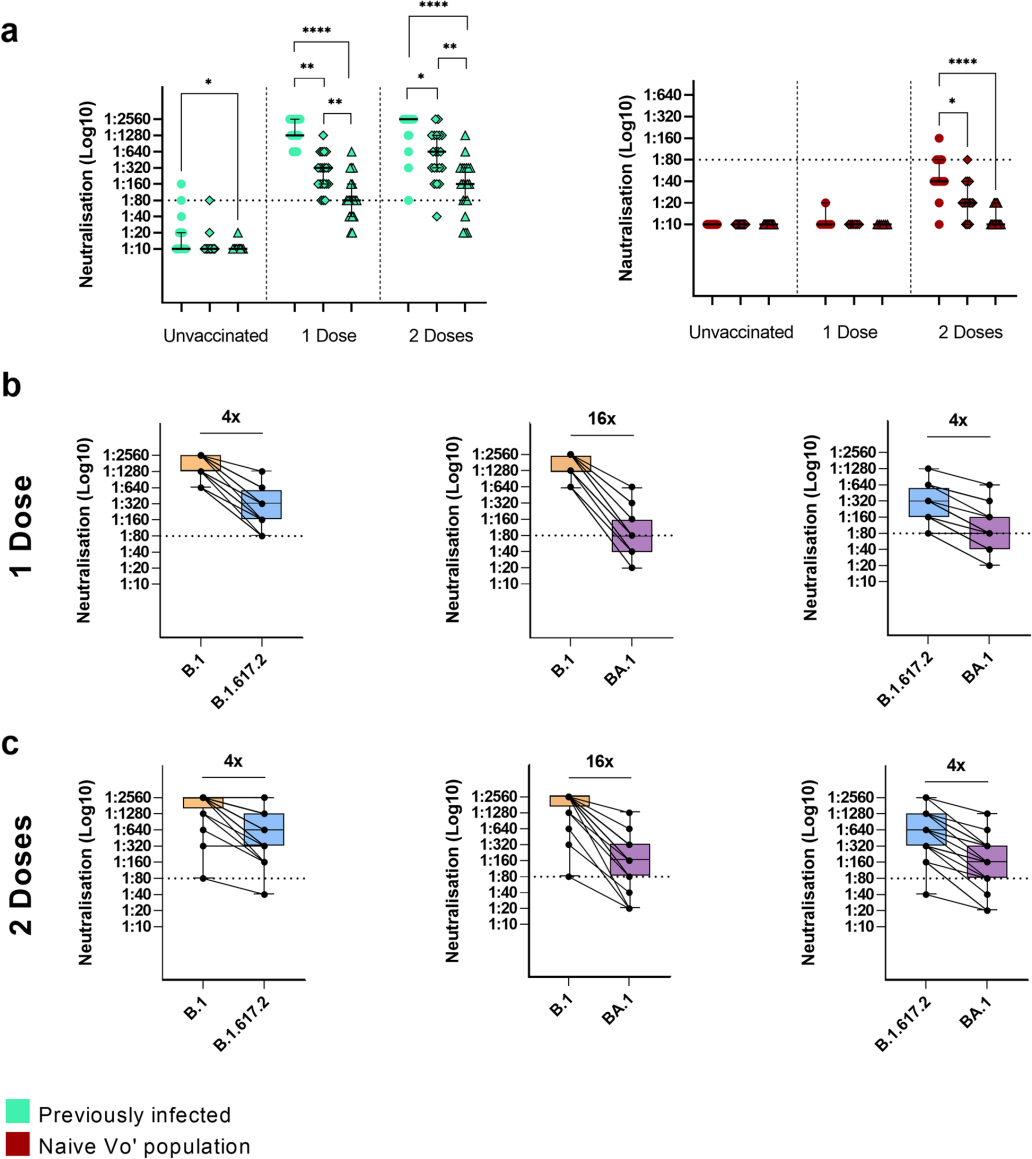
Neutralisation titres against the B.1, B.1.617.2 and BA.1 SARS-CoV-2 variants in naïve and previously infected subjects according to vaccination. **a** Neutralising antibody titres against B.1, B.1.617.2 and BA.1 SARS-CoV-2 variants in (left) previously infected (Friedman test followed by Dunn’s multiple comparisons test, serum of previously infected subjects with one vaccine dose against B.1 versus B.1.617.2 and BA.1, *P* = 0.004 and *P* < 0.0001 respectively; serum of previously infected subjects with two vaccine doses against B.1 versus B.1.617.2 and BA.1, *P* = 0.02 and *P* < 0.0001, respectively) and (right) naïve subjects (Friedman test followed by Dunn’s multiple comparisons test, serum of naïve subjects with two doses of vaccine against B.1 versus B.1.617.2 and BA.1, *P* = 0.03 and *P* <0.0001, respectively). Asterisks indicate *P < 0.05, **P < 0.01, ***P < 0.001, ****P < 0.0001. **b**, **c** Fold changes in serum neutralisation titres of previously infected subjects with **b** one dose of vaccine and **c** two doses of vaccine tested for B.1.617.2 and BA.1 variants compared to B.1 (fold change of 4× and 16× for both one or two doses of vaccine for B.1.617.2 and BA.1, respectively). Previous infection is defined according to the ground truth definition given in the text

The sera obtained from vaccinated individuals previously infected by SARS-CoV-2 and subjects vaccinated when naïve were tested in a micro-neutralisation assay against the B.1.617.2 and BA.1 variants, to assess the neutralising ability of the humoral immunity mounted upon vaccination. Lower neutralising titres against the B.1.617.2 and BA.1 than compared to the B.1 strain were observed in individuals vaccinated after natural infection, both after one and two vaccine doses (Friedman test followed by Dunn’s multiple comparisons test, B.1 neutralisation compared to B.1.617.2 after 1 and 2 doses of vaccine *P* = 0.004 and *P* = 0.02 respectively, B.1 neutralisation compared to BA.1 after 1 and 2 doses of vaccine *P* <0.0001 for both cases). Nevertheless, all but one subject (97.5%, 95% CI 86.8–99.9%) maintained a neutralisation titre >1:40 (1/dil) against B.1.617.2 strain (20 out of 20 after one dose of vaccine, 100%, 95% CI 83.2–100%, 19 out of 20 after two doses of vaccine, 95%, 95% CI 75.1–99.9%) (Fig. 6a, left panel) and 75.0% (30 out of 40, 95% CI 58.8–87.3%) had titre >1:40 (1/dil) against BA.1 strain (14 out of 20 after one dose of vaccine, 70%, 95% CI 45.7–88.1%, 16 out of 20 after two doses of vaccine, 80%, 95% CI 56.3–94.3%). The decrease in neutralisation caused by the B.1.617.2 and BA.1 variants was observed also in unvaccinated individuals previously infected (Mann-Whitney test, *P* < 0.001), but in a context where most of them displayed low neutralising titres also against the B.1 strain (33 out of 35 (94.3%, 95% CI 80.8–99.3%), 34 out of 35 (97.1%, 95% CI 85.1–99.9%), and 35 out of 35 (100%, 95% CI 90–100%) subjects with neutralising titres below 1:80 (1/dil) threshold against the B.1, B.1.617.2, and BA.1 variants, respectively). A similar trend was present in subjects vaccinated when naïve (Fig. 6a right panel) (Friedman test followed by Dunn’s multiple comparisons test, *P* = 0.03 and *P* < 0.0001 after two vaccine doses for B.1.617.2 and BA.1 variants, respectively). A difference of four- and sixteen-fold changes was observed in previously infected subjects with at least one dose of vaccine when comparing the median neutralisation titres for B.1 with B.1.617.2 and BA.1, respectively.

## Discussion

Monitoring the serological response to SARS-CoV-2 infection and vaccination over time is crucial to estimate the persistence of circulating antibodies, their neutralising efficacy, and to inform vaccination policies. Due to the continuous emergence of new viral variants [35], it is critical to assess the extent to which previous immunity, developed from natural infection or vaccination, protects against the new circulating strains.

The Vo’ cohort is a highly characterised population including a core of individuals identified as infected by SARS-CoV-2 back in February/March 2020, which has been followed up through time in sequential swab and serological surveys until June 2021, roughly 15 months after viral exposure, thus offering unique insights into the long-term antibody dynamics. The results presented in this study confirm the trends observed in our previous follow-up, performed at 9 months since the first wave in Vo’ [8], with strong variability observed among serological tests, especially for the two assays targeting the N viral antigen.

Of the identified SARS-CoV-2 cases who acquired the infection in February/March 2020, only 11.8% (95% CI 5.6–21.3%) tested positive by Abbott while 93.4% (95% CI 85.3–97.8%) tested positive by Roche after 15 months. This discrepancy was already reported in other studies [36–38] and could be due to the use of an antigen bridging approach in the Roche assay, where declines in the total amount of antibody might be compensated by increases in affinity or avidity as antibodies mature through somatic hypermutation, to differences in the employed antigens, and to the fact that the N epitopes recognised by antibodies might change with time. In perspective, given that these two tests could allow to discern recent from past infections, they could be employed in future seroprevalence studies to assess the attack rate in vaccinated subjects and thus provide new data on the frequency of breakthrough infections as well as re-infection.

We found that all individuals infected at the start of the pandemic and tested 15 months later are positive to at least one serological assay, although the decreasing trend of antibody levels against both S and N antigens is confirmed, independently from the type of test used. In the absence of vaccination, the neutralising titres of the infected subjects drop almost completely below the 1:80 (1/dil) threshold.

While the observed decrease in antibody titres is in line with other recent reports [8–11], it does not necessarily translate into an impaired immunity in these subjects, since humoral response is one arm of the adaptive immune response, which also includes cellular immunity and reactivation upon stimulation of memory B and T cells [17, 39, 40].

Unexpectedly, we found a significant difference in the amount of circulating N targeting antibodies between vaccinated and unvaccinated subjects previously infected by SARS-CoV-2. To investigate this observation, we retrospectively analysed the differences in N targeting antibodies present in the two groups in the November 2020 serosurvey, before the beginning of the vaccination campaign. We found that the two groups are significantly different in terms of age, with vaccinated subjects being older than unvaccinated subjects (Additional file 1: Fig. S2e). The age difference can be explained by the vaccination strategy and agrees with our previous observation that antibody levels were higher with increasing age in this cohort [8].

We found that the response to vaccination is different among subjects vaccinated after infection or when naïve: while a marked increase in S-targeting antibodies is observed in all individuals, antibodies induced by vaccination are higher in previously infected subjects. In vaccinated previously infected subjects, a single dose of vaccine is shown to boost a strong neutralisation response, as confirmed in other studies [18, 41]. This suggests that a single dose of vaccine in previously infected individuals induces a robust immune response in support of the vaccination strategy implemented in Germany, France, Italy, and Israel among other countries. It has been shown that B cell maturation due to somatic hypermutation, possibly stimulated by long-term persistence of viral antigens in specific body niches [12, 42–44], can produce stronger and more specific antibodies [45].

By comparing the antibody levels in vaccinated naïve subjects (in June 2021) with those of patients who recovered from natural infection in May 2020, we demonstrate that a complete vaccination course confers stronger immunity than natural infection alone, at least in terms of serum antibodies as detected by both DiaSorin and neutralisation.

Finally, we tested the ability of antibodies developed against SARS-CoV-2 strains circulating early in the pandemic to neutralise both the Delta and Omicron variants of concern (VOC B.1.617.2 and BA.1, respectively), which are characterised by several mutations in the spike protein and an increased transmissibility that allowed them to become prevalent worldwide at different stages of the pandemic [46]. We observed a decrease in neutralising reactivity across all immunity profiles (infected and unvaccinated, infected and vaccinated, and vaccinated when naïve), although with substantial differences in terms of magnitude. In fact, whereas the absolute reduction in neutralising reactivity is larger in subjects vaccinated after having being infected (i.e. those with hybrid immunity, Fig. 6a, left panel), most of them maintained a neutralising titre above 1:40 (1/dil) for both Delta (97.5%) and Omicron (75%) variants. On the contrary, sera from naïve vaccinated subjects showed a more limited reduction in neutralising titres when challenged with Delta and Omicron variants, but almost all of them showed titres below 1:40 (1/dil). The molecular basis of the increased protection conferred by hybrid immunity is not completely understood, but recent findings on B-cell repertoires at the single-cell level identified the expansion of a specific germline, IGHV2-5; IGHJ4-1, which was expanded only in subjects vaccinated after natural infection but not in naive vaccines and was associated with potent and broadly neutralising antibodies [21]. In this regard, a third vaccine dose administered to subjects who received their first course of vaccination when naïve could mimic hybrid immunity, also by promoting affinity maturation and higher avidity of antibodies [47–49].

Since neutralising IgG antibodies are the best current indication for protection against reinfection and correlate well with virological response and survival [19, 50], these findings are of particular importance in consideration of the efforts and resources that have been invested in the vaccination campaign in Italy and worldwide.

## Conclusions

Our results show that the vaccines currently deployed in Europe, although developed on a viral strain that is no longer circulating, are essential and effective in conferring protection against currently circulating variants (as of April 2022), although two doses are not sufficient to induce strong neutralising reactivity versus Delta and Omicron variants in naïve individuals. These results confirm that vaccination is a safe and effective strategy to generate immunity against SARS-CoV-2, but also that the new variants are rapidly escaping immune recognition. While it is critical to maintain and strengthen epidemiological and genomic surveillance to monitor the potential emergence of new immune-escaping variants, the control of the present and potential future coronavirus pandemics will be achieved with the development of new pan-coronavirus vaccines generating a wide protection against future emerging SARS-CoV-2 variants and new pathogens belonging to the same viral family [51].

## Supplementary Information

**Additional file 1: Figure S1.** Anti-N antibody titres and dynamics in vaccinated and unvaccinated subjects; **Figure S2.** Anti-N antibody titres of vaccinated and unvaccinated individuals previously exposed to SARS-CoV-2 infection differ according to age; **Figure S3.** Correlation among DiaSorin S1/S2, DiaSorin trimeric tests and neutralisation assay; **Figure S4.** Features of the cohorts analysed in this study. **Table S1.** Concordance between S1/S2 and trimericS DiaSorin assays.

## Data Availability

All the data and metadata related to the current study, the source code that calculates the antibody and the neutralisation half-life, and the source code that calculates antibody titre association to metadata are available at https://github.com/MedCompUnipd/Vo-Serology.git [52].

## Abbreviations

SARS-CoV-2: Severe acute respiratory syndrome coronavirus 2
COVID19: Coronavirus disease 2019
EMA: European Medicines Agency
FDA: Food and Drug Administration
VOC: Variant of concern
S: Spike
N: Nucleocapsid
CI: Confidence interval
GT: Ground truth, infected Vo’ population
HCW: Healthcare workers
