# Supplementary material for: Neutralising reactivity against SARS-CoV-2 Delta and Omicron variants by vaccination and infection history
Source: Genome Med. 2022 Jun 10;14:61. doi: 10.1186/s13073-022-01066-2 (PMC9185135; doi:10.1186/s13073-022-01066-2)
Supplement: Supplementary file 1 — Additional file 1: Figure S1. Anti-N antibody titres and dynamics in vaccinated and unvaccinated subjects; Figure S2. Anti-N antibody titres of vaccinated and unvaccinated individuals previously exposed to SARS-CoV-2 infection differ according to age; Figure S3. Correlation among DiaSorin S1/S2, DiaSorin trimeric tests and neutralisation assay; Figure S4. Features of the cohorts analysed in this study. Table S1. Concordance between S1/S2 and trimericS DiaSorin assays. [file 13073_2022_1066_MOESM1_ESM.docx]

**Neutralising reactivity against SARS-CoV-2 Delta and Omicron variants by vaccination and infection history**

Enrico Lavezzo^1^^, Monia Pacenti^2^^, Laura Manuto^1^^, Caterina Boldrin^2^, Margherita Cattai^2^, Marco Grazioli^1^, Federico Bianca^1^, Margherita Sartori^1^, Federico Caldart^3^, Gioele Castelli^4^, Michele Nicoletti^4^, Eleonora Nieddu^5^, Elisa Salvadoretti^6^, Beatrice Labella^7^, Ludovico Fava^4^, Maria Cristina Vanuzzo^2^, Vittoria Lisi^2^, Maria Antonello^1^, Carmela Ileana Grimaldi^1^, Chiara Zulian^2^, Claudia Del Vecchio^1^, Mario Plebani^8^, Andrea Padoan^8^, Daniela Cirillo^9^, Alessandra R Brazzale^10^, Giovanni Tonon^11,12^, Stefano Toppo^1,^*, Ilaria Dorigatti^13,^* & Andrea Crisanti^1,2,14,^*

^Contributed equally

* Joint senior authors

^1^Department of Molecular Medicine, University of Padova, Padova, Italy

^2^Azienda Ospedale Padova, Padova, Italy

^3^Gastroenterology Unit, Department of Medicine, Verona B. Roma University Hospital, Verona, Italy

^4^Department of Cardiac, Thoracic, Vascular Sciences and Public Health, University of Padova, Padova, Italy

^5^Department of Surgery, Oncology and gastroenterology, University of Padova, Padova, Italy

^6^Paediatrics Unit, Mother and Child Hospital, Surgery, Dentistry, Maternity and Infant Department, Verona University Hospital, Verona, Italy

^7^Neurology Unit, Department of Clinical and Experimental Sciences, University of Brescia, Brescia, Italy

^8^Department of Medicine, University of Padova, Padova, Italy

^9^Emerging Bacterial Pathogens Unit, Division of Immunology, Transplantation and Infectious Diseases, IRCCS San Raffaele Scientific Institute, Milan, Italy

^10^Department of Statistical Sciences, University of Padova, Padova, Italy

^11^Center for Omics Sciences, IRCCS Ospedale San Raffaele, Milan, Italy

^12^Functional Genomics of Cancer Unit, Division of Experimental Oncology, IRCCS San Raffaele Scientific Institute, Milano, Italy

^13^MRC Centre for Global Infectious Disease Analysis and Jameel Institute, School of Public Health, Imperial College London, London, United Kingdom

^14^Department of Life Sciences, Imperial College London, London, UK


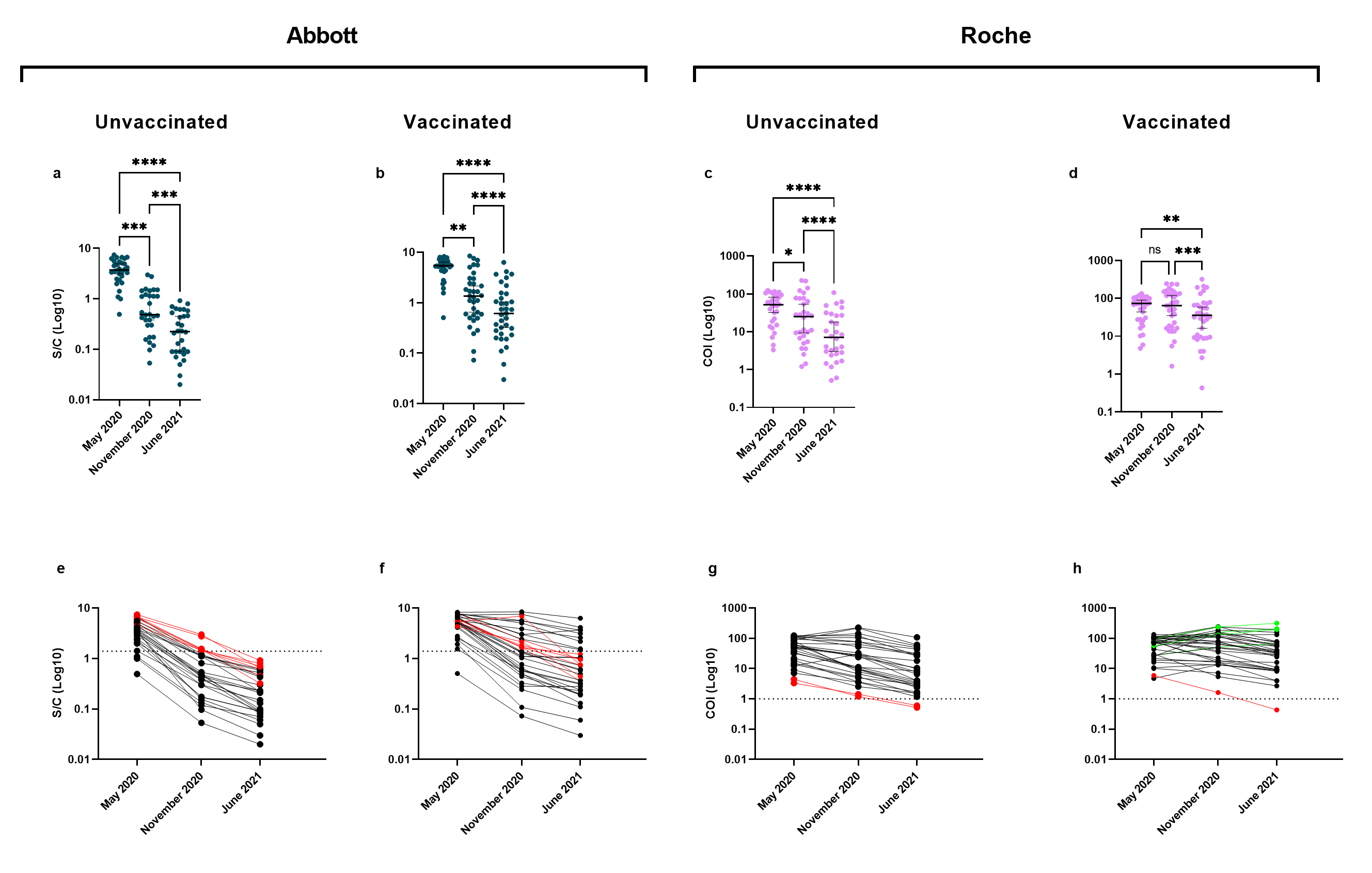


**Fig. S1.** **Anti-N antibodies titres and dynamics in vaccinated and unvaccinated subjects**

Observed antibody titres in vaccinated and unvaccinated subjects who were previously exposed to SARS-CoV-2 and tested in May 2020, November 2020, and June 2021 by Abbott and Roche assays. Vaccines have no impact on anti-N antibody titres, which decreased for 100% (65 out of 65 subjects, 95% CI 94.5-100%, p<0.0001 from November 2020 to June 2021) and 93.8% (61 out of 65 subjects, 95% CI 85.0-98.3%, p=0.004 from November 2020 to June 2021) according to Abbott and Roche assays, respectively. Asterisks indicate *p < 0.05, **p < 0.01, ***p < 0.001, ****p < 0.0001. Statistical significance was evaluated by Friedman test followed by Dunn's multiple comparisons test.


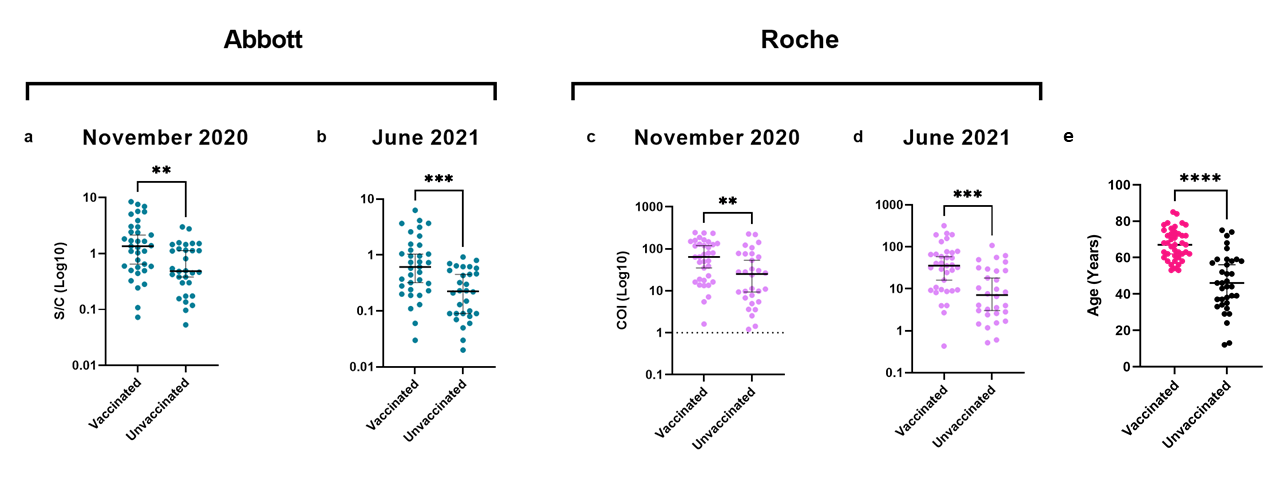


**Fig. S2. Anti-N antibody titres of vaccinated and unvaccinated individuals previously exposed to SARS-CoV-2 infection differ according to age.** a-d) Anti-N antibody titres of vaccinated and unvaccinated subjects pre-exposed to SARS-CoV-2 by Abbott (Mann-Whitney test, p=0.0037 AND P=0.0005 for November 2020 and June 2021, respectively) and Roche assays (Mann-Whitney test, p=0.0058 and p=0.0003 for November 2020 and June 2021, respectively). e) Age of vaccinated and unvaccinated individuals with past exposure to SARS-CoV-2 (Mann-Whitney test, p<0.0001).


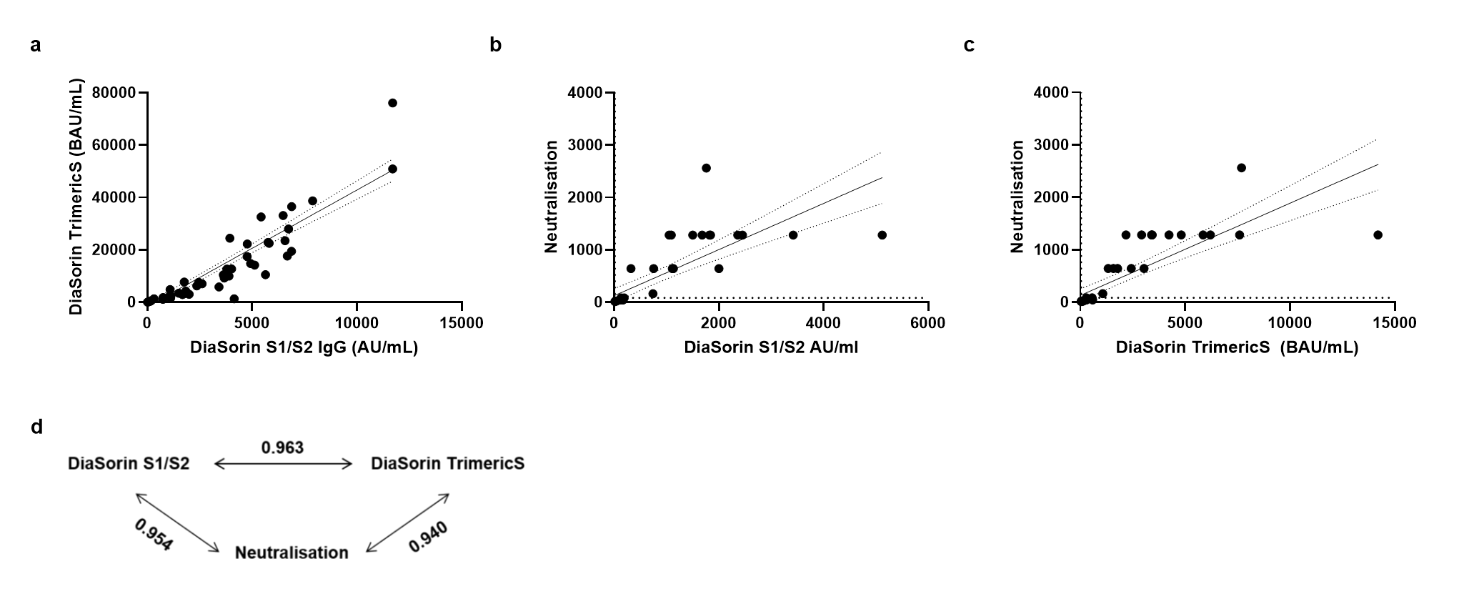


**Fig. S3. Correlation among DiaSorin S1/S2, DiaSorin trimeric tests and neutralisation assay.** a-d) Spearman’s correlation among DiaSorin S1/S2, DiaSorin TrimericS and micro-neutralisation assays. DiaSorin S1/S2 vs Diasorin TrimericS: Spearman’s r = 0.963, 95% CI 0.941-0.977; DiaSorin S1/S2 vs neutralisation: Spearman’s r = 0.954, 95% CI 0.916-0.975; DiaSorin TrimericS vs neutralisation: Spearman’s r = 0.940, 95% CI 0.890-0.967; all p values are significant, P < 0,0001.


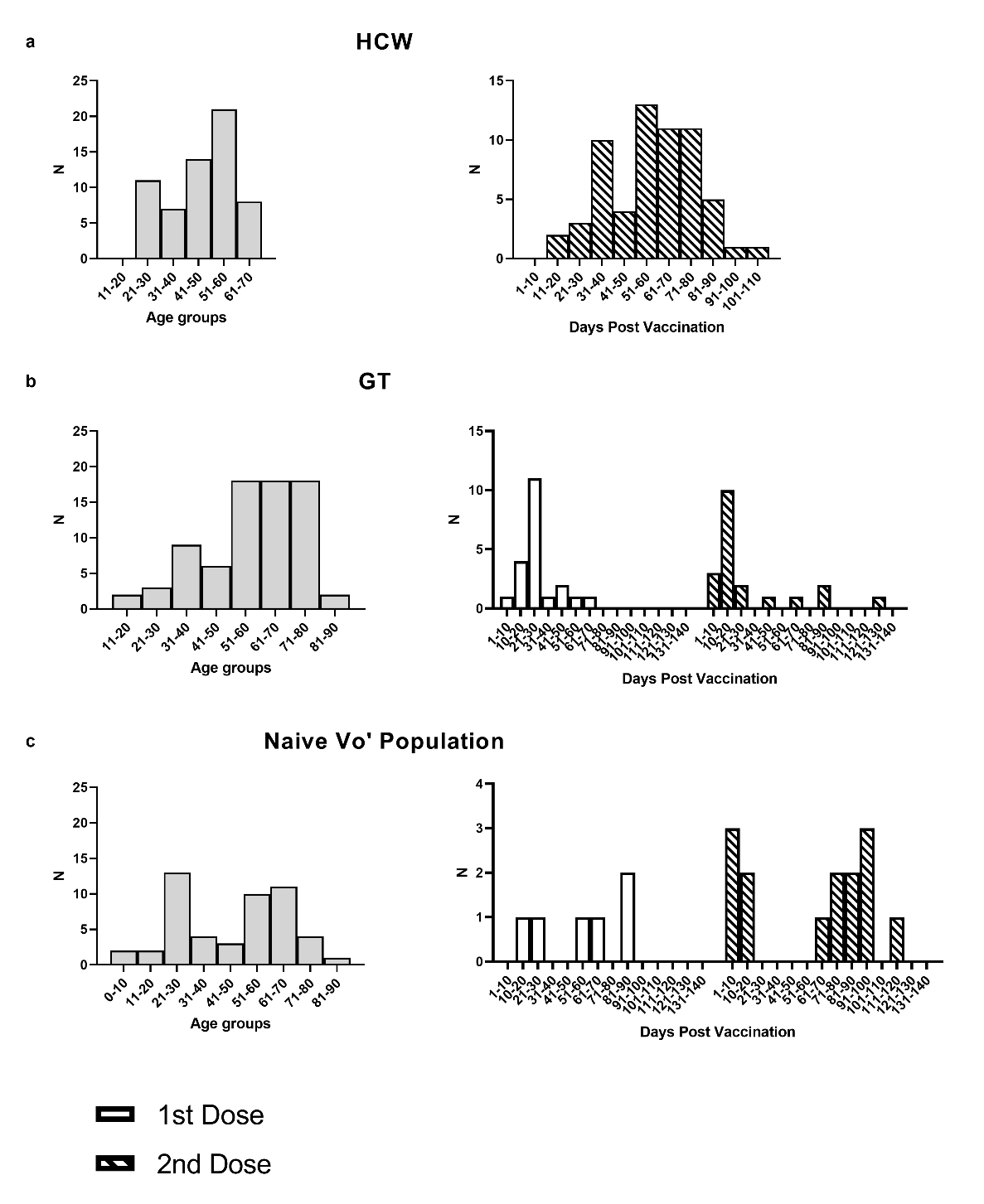


**Fig. S4. Features of the cohorts analysed in this study.** Age distribution and distance between vaccination and serological test in the (a) healthcare workers (HCW)(median age 48 years, median days post-vaccination (two doses) 60 days), (b) Vo’ pre-exposed subjects (ground truth, GT)(median age 60.5 years, median days post-first dose 25 days, median days post-second dose 17 days), and (c) Vo’ naïve subjects (median age 54 years, median days post-first dose 62.5 days, median days post-second dose 96 days) cohorts.

**Table S1: Concordance between S1/S2 and trimericS DiaSorin assays**

| **n = 76 tested in June 2021 and belonging to the ground truth** | | |
| --- | --- | --- |
|  | **DiaSorin S1/S2 IgG+** | **DiaSorin TrimericS IgG+** |
| **DiaSorin S1/S2 IgG+** | 61 | 61 |
| **DiaSorin TrimericS IgG+** |  | 68 |
